# Laboratory parameters related to disease severity and physical performance after reconvalescence of acute COVID-19 infection

**DOI:** 10.1038/s41598-024-57448-6

**Published:** 2024-05-06

**Authors:** Mario Gietl, Francesco Burkert, Stefanie Hofer, Johanna M. Gostner, Thomas Sonnweber, Ivan Tancevski, Alex Pizzini, Sabina Sahanic, Andrea Schroll, Natascha Brigo, Alexander Egger, Rosa Bellmann-Weiler, Judith Löffler-Ragg, Günter Weiss, Katharina Kurz

**Affiliations:** 1 Department of Internal Medicine II, Medical University of Innsbruck, Anichstrasse 35, 6020 Innsbruck, Austria; 2 Institute of Medical Biochemistry, Biocenter, Medical University of Innsbruck, Innrain 80, 6020 Innsbruck, Austria; 3 Central Institute for Medical and Chemical Laboratory Diagnostics (ZIMCL), Tirol Kliniken GmbH, Anichstrasse 35, 6020 Innsbruck, Austria

**Keywords:** Infectious diseases, Immunology

## Abstract

Research into the molecular basis of disease trajectory and Long-COVID is important to get insights toward underlying pathophysiological processes. The objective of this study was to investigate inflammation-mediated changes of metabolism in patients with acute COVID-19 infection and throughout a one-year follow up period. The study enrolled 34 patients with moderate to severe COVID-19 infection admitted to the University Clinic of Innsbruck in early 2020. The dynamics of multiple laboratory parameters (including inflammatory markers [C-reactive protein (CRP), interleukin-6 (IL-6), neopterin] as well as amino acids [tryptophan (Trp), phenylalanine (Phe) and tyrosine (Tyr)], and parameters of iron and vitamin B metabolism) was related to disease severity and patients’ physical performance. Also, symptom load during acute illness and at approximately 60 days (FU1), and one year after symptom onset (FU2) were monitored and related with changes of the investigated laboratory parameters: During acute infection many investigated laboratory parameters were elevated (e.g., inflammatory markers, ferritin, kynurenine, phenylalanine) and enhanced tryptophan catabolism and phenylalanine accumulation were found. At FU2 nearly all laboratory markers had declined back to reference ranges. However, kynurenine/tryptophan ratio (Kyn/Trp) and the phenylalanine/tyrosine ratio (Phe/Tyr) were still exceeding the 95th percentile of healthy controls in about two thirds of our cohort at FU2. Lower tryptophan concentrations were associated with B vitamin availability (during acute infection and at FU1), patients with lower vitamin B12 levels at FU1 had a prolonged and more severe impairment of their physical functioning ability. Patients who had fully recovered (ECOG 0) presented with higher concentrations of iron parameters (ferritin, hepcidin, transferrin) and amino acids (phenylalanine, tyrosine) at FU2 compared to patients with restricted ability to work. Persistent symptoms at FU2 were tendentially associated with IFN-γ related parameters. Women were affected by long-term symptoms more frequently. Conclusively, inflammation-mediated biochemical changes appear to be related to symptoms of patients with acute and Long Covid.

## Introduction

COVID-19 infection, caused by severe acute respiratory syndrome coronavirus 2 (SARS-CoV-2), typically manifests as a mild infection, but can also lead to a severe and potentially fatal illness^1^. A malfunctioning systemic immune response is linked to severe illness and poorer patient outcomes^2–4^.

Studies have shown markedly elevated levels of inflammatory markers like IL-6 in COVID-19 infected patients^5–7^. Meta-analyses demonstrated that patients with severe COVID-19 infection had higher levels of inflammatory markers and acute phase proteins like CRP, procalcitonin (PCT), IL-6, and serum ferritin compared to patients with a mild disease course^8–11^.

After numerous studies on HIV^12–14^ and cancer^15,16^, recent studies have confirmed that interferon gamma (IFN-γ) mediated Trp catabolism and neopterin formation are also strongly induced in patients with COVID-19 infection^17–20^. Accordingly, Trp catabolism was shown to be predictive for a worse COVID-19 outcome in recent literature^20,21^. Symptoms like depression and impaired quality of life have also been proposed to be exacerbated by IFN-γ induced indoleamine 2,3-dioxygenase 1 (IDO1): as the subsequent degradation of Trp along the kynurenine pathway reduces the formation of serotonin, which can go along with heightened anxiety, cognitive deficits, and sleep disturbances^22–26^. Accordingly, low Trp levels have been linked to sleep disturbance during acute COVID-19 infection^27^ and persistence of symptoms (Long-COVID)^28^.

Apart from Trp catabolism, also other inflammation-mediated processes might impair patients' physical and mental abilities during and after infection: Th1 type immune response induces perturbations of iron metabolism^29^ and goes along with increased oxidation of B vitamins^30^. Both metabolic changes might contribute importantly to the development of fatigue: Anemia and disturbed iron metabolism have been demonstrated to be linked with poor physical performance in COVID-19 patients with persisting symptoms^31^. Additionally, B vitamin deficiency can contribute to anemia and neuro-psychiatric symptoms^32,33^.

Vitamin B12, also known as cobalamin, plays an important role in viral infections as well as in systemic inflammation^34,35^. A study showed that surviving COVID-19 infected individuals had higher levels of vitamin B12 compared to those who died from the virus^36^. Lower vitamin B12 also seems to be related to smell affection in COVID-19 patients^37^. According to a recent transcriptome analysis, vitamin B12 blocks several inflammation-related pathways that are activated in patients with moderate or severe COVID-19 infection^38^. However, there are also studies, which report an inverse relationship^39^ or no link between COVID-19 severity and vitamin B12 concentrations^40^.

In this study we investigated IFN-γ related biochemical parameters and routine laboratory values like vitamin B12 as well as disease severity and patients' performance status measured by the Eastern Cooperative Oncology Group (ECOG) scale in moderate to severe COVID-19 infected individuals over the course of two follow-ups within a one-year period.

## Materials and methods

### Participants and study design

This study included 34 patients (25 men, 9 women, 62.0 years median age) who were hospitalized for treatment of acute COVID-19 infection from March to May 2020. Follow-up assessments were conducted at approximately 60 days (FU1) and one year (FU2) after the onset of symptoms. All patients came to the outpatient department for FU1 and 28 patients for FU2 (21 men, 7 women). These assessments involved blood sampling, a questionnaire, and a medical examination for each patient. All patients gave repeated written and informed consent for their data to be used for scientific purposes and the studies were approved by the ethical board of the Medical University of Innsbruck (1167/2020 and 1157/2017). The study was performed in accordance with the Declaration of Helsinki.

The fact that hypothesis driven blood sampling was not routinely performed at the beginning of the pandemic accounts for missing values in patient data.

### Laboratory analysis

At the first or second day of hospitalization, at FU1 and at FU2 the routine laboratory values were determined by the ISO 15,189 accredited Central Institute for Medical and Chemical Laboratory Diagnostic (ZIMCL) in Innsbruck, Austria as follows: CRP, IL-6, PCT, iron, transferrin, ferritin, folate, vitamin B12, creatine kinase (CK), troponin T, NT-proBNP, serum glutamic oxaloaxetic transaminase (GOT), serum glutamic pyruvic transaminase (GPT), and lactate dehydrogenase (LDH) were analysed on a Cobas 8000 platform (Roche Diagnostics, Rotkreuz, Switzerland). Bioactive Hepcidin was determined by ELISA (DRG Instruments GmbH, Marburg, Germany) on a BEP2000, and 25-OH Vit D was measured by HPLC using the kit from Chromsystems Instruments and Chemicals GmbH (Graefelfing, Germany). All hematologic parameters were determined by an XN-2000 analyzer from Sysmex (Kobe, Japan).

Aromatic amino acids and the Trp derivative kynurenine (Kyn), as well as neopterin were analyzed at the Institute of Medical Biochemistry, Biocenter, Medical University of Innsbruck. The quantification of aromatic amino acids and Kyn as catabolite of Trp was performed as described previously^27^. Neopterin concentrations were measured by ELISA (BRAHMS Diagnostica, Berlin, Germany), nitrite was determined by Griess reaction^41^. The ratios of kynurenine/tryptophan (Kyn/Trp) and phenylalanine/tyrosine (Phe/Tyr) were calculated as a surrogate of IDO-1^42^ and phenylalanine hydroxylase (PAH)-activity^43^.

All methods were performed in accordance with the relevant guidelines and regulations.

### Questionnaire

The questionnaire, administered at multiple follow-up time points (FU1 and FU2) included questions about Eastern Cooperative Oncology (ECOG) score and sleep impairment. Acute COVID-19 infection data was collected retrospectively at FU1. The presence of sleep disturbance was determined either by the patient mentioning it or if there was prescription of sleep medication.

### Additional data and definitions

Neurological symptoms were documented based on the patients' medical records. Fatigue data was gathered through the structured medical interview. COVID-19 severity were categorized based on the need for medical treatment, in accordance with the classification used by Sonnweber et al^44^. Mild severity was defined as outpatient treatment, moderate severity as inpatient treatment without respiratory support, severe disease as inpatient treatment with oxygen therapy, and critical disease as the need for mechanical ventilation in an intensive care unit (ICU).

### Statistical analysis

Descriptive statistics include categorical variables, which were displayed as frequencies and percentages, and non-normally distributed continuous variables, which were assessed using median and range. Most of the variables were not normal distributed; therefore, non-parametric tests were applied. Mann–Whitney U-test was used to compare two independent groups, Wilcoxon signed rank test yielded results for two dependent groups, and Kruskal–Wallis test was used for comparison of more than two independent groups. Post-Hoc tests for multiple comparisons included Bonferroni correction to counteract alpha error accumulation. Correlations were evaluated using Spearman rank test. The significance level was set to *p* < 0.05. All analyses were performed using IBM SPSS Statistics 28.0 (IBM Corp., USA).

## Results

### Baseline characteristics

34 patients who had been hospitalized for COVID-19 infection came for FU1, 28 of them also for FU2 (one year after infection). The median age was 62 years (interquartile range (IQR) 54–71) ranging from 44 to 87 years. 73.5% (male, n = 25) and 26.5% (female, n = 9). Three patients (8.8%) had a moderate severity, eleven patients (32.4%) had a severe disease, and 20 patients (58.8%) were admitted to the ICU during treatment for acute COVID-19 infection, representing a critical outcome. No patient in the study died because of SARS-CoV-2 infection. Median age was 60.0 years in the moderate group, 72.0 years in the severe group and 57.5 years in the ICU group. Regarding sex, statistical testing yielded no significant difference between men and women for disease severity (p = 0.561). During acute COVID-19 infection 19 out of the 34 patients of our sample reported an ECOG of 4, representing a total inability to care for themselves. Four patients reported an ECOG of 3, six patients reported an ECOG of 2, three patients reported an ECOG of 1, and one patient reported an ECOG of 0. At FU1 12 patients reported an ECOG of 0 and 1 respectively, while an ECOG of 2 was reported in five patients and an ECOG of 4 in one patient. About one year after infection 14 patients had an ECOG of 0, 13 patients an ECOG of 1 and one patient an ECOG of 2. ECOG-Score was significantly higher during acute illness compared to FU1 (*p* < 0.001) and FU2 (*p* < 0.001).

During the hospital stay for acute COVID-19 infection the percentage of men and women reporting symptoms like fatigue, sleep disturbance and neurological symptoms was comparable. However, on average, women were more frequently suffering from persisting symptoms in the reconvalescence phase (FU1 and FU2; see Table 1). 62.5% of female patients were reporting neurological symptoms compared to 14.3% of men at FU2. This was surprising, as no woman had reported neurological symptoms at FU1. Sleep disturbance was prevalent in three out of four women, compared to 35% of male patients also at FU2. Sleep disturbance had already been more frequent in women at FU1 (62.5% women versus 27.3% of men). Moreover, half of the female patients were fatigued at FU2 and 23.8% of men.

**Table 1:** Frequencies of nominal study parameters during acute COVID-19 and at the two follow-ups.

		Neurological symptoms		Fatigue		Sleep disturbance	
		n (valid %)	missing	n (valid %)	missing	n (valid %)	missing
**Acute**	**Total**	**18 (52.9)**	**0**	**33 (100)**	**1**	**20 (60.6)**	**1**
	Male	13 (52.0)	0	24 (100)	1	15 (60.0)	0
	Female	5 (55.6)	0	9 (100)	0	5 (62.5)	1
**FU1**	**Total**	**6 (20.0)**	**4**	**16 (53.3)**	**4**	**11 (36.7)**	**4**
	Male	6 (27.3)	3	11 (50.0)	3	6 (27.3)	3
	Female	0 (0)	1	5 (62.5)	1	5 (62.5)	1
**FU2**	**Total**	**8 (27.6)**	**5**	**9 (31.0)**	**5**	**13 (46.4)**	**6**
	Male	3 (14.3)	4	5 (23.8)	4	7 (35.0)	5
	Female	5 (62.5)	1	4 (50.0)	1	6 (75.0)	1

n, number of patients; missing, number of patients with missing value; valid %, percentage of patients excluding missing values; n = 34 during acute illness; n = 34 at FU1; n = 28 at FU2.

Significant values are in [bold].

### Laboratory parameters

Several parameters differed significantly between acute COVID-19 infection and the two follow-ups (FU1–FU2) (see Table 2 for sex-stratified values).

**Table 2:** Sex-stratified study parameters and comparison of acute COVID-19 infection (acute) and FU1–FU2.

	Gender	Acute		FU1		FU2
		Median (IQR)	*p*-value*	meDian (IQR)	*p*-value **	Median (IQR)
Neopterin [nmol/L]	Male	**26.3 (17.7–44.4)**	** < 0.001**	**8.9 (8.0–13.0)**	n.s	7.8 (7.1–9.3)
	Female	**41.1 (18.3–45.7)**	**0.038**	**12.5 (9.1–18.9)**	0.069	6.5 (5.8–8.9)
Kyn [µmol/L]	Male	**3.1 (2.3–3.8)**	**0.049**	**2.3 (1.8–3.0)**	n.s	2.5 (2.2–2.7)
	Female	3.5 (3.1–4.0)	0.051	2.4 (2.2–2.5)	n.s	2.3 (2.0–3.1)
Trp [µmol/L]	Male	**45.4 (40.8–52.4)**	**0.040**	**52.8 (48.2–59.8)**	0.061	62.0 (58.8–69.3)
	Female	41.2 (35.7–51.5)	n.s	**54.4 (40.8–54.9)**	**0.012**	**65.9 (60.9–70.6)**
Phe [µmol/L]	Male	**125.3 (114.2–134.8)**	** < 0.001**	**69.9 (64.4–87.2)**	n.s	84.1 (73.4–92.5)
	Female	**129.9 (113.7–157.1)**	**0.008**	**60.9 (56.4–69.3)**	**0.025**	**73.3 (68.7–95.1)**
Tyr [µmol/L]	Male	66.3 (58.9–72.5)	n.s	**58.3 (54.0–67.2)**	**0.020**	**81.0 (66.8–92.2)**
	Female	62.6 (57.9–73.4)	n.s	**59.8 (57.3–72.9)**	**0.012**	**78.3 (68.3–91.0)**
Kyn/Trp [µmol/mmol]	Male	**63.8 (51.2–83.4)**	**0.006**	**39.3 (33.7–55.5)**	n.s	37.6 (36.3–44.1)
	Female	**82.6 (61.4–96.9)**	**0.028**	**45.5 (41.0–56.8)**	**0.025**	**36.0 (32.3–44.5)**
Phe/Tyr [µmol/µmol]	Male	**1.9 (1.7–2.1)**	** < 0.001**	**1.2 (1.0–1.4)**	0.088	1.0 (0.9–1.1)
	Female	**2.0 (1.6–2.3)**	**0.008**	**1.0 (1.0–1.1)**	n.s	1.0 (0.9–1.0)
Nitrite [µmol/L]	Male	23.3 (13.8–45.4)	n.s	**29.6 (11.8–55.6)**	**0.005**	**8.8 (8.4–17.2)**
	Female	22.4 (13.3–33.2)	n.s	**16.5 (10.7–52.4)**	**0.036**	**7.7 (5.7–14.9)**
Folate [µg/L]	Male	**10.3 (5.3–11.9)**	**0.048**	**5.6 (4.5–8.3)**	**0.028**	**7.7 (6.1–10.8)**
	Female	15.4 (13.1–17.6)	n.s	7.5 (4.9–16.6)	n.s	16.9 (10.3–20.0)
Vitamin B12 [pmol/mL]	Male	**376 (268–493)**	**0.026**	**234 (188–327)**	n.s	282 (217–395)
	Female	604 (374–826)	n.s	352 (311–535)	n.s	383 (302–581)
WBC [G/L]	Male	6.0 (3.6–7.4)	n.s	6.3 (5.3–7.4)	n.s	6.9 (5.9–7.7)
	Female	6.5 (6.1–10.4)	n.s	**7.1 (6.5–8.4)**	**0.018**	**5.7 (5.3–7.2)**
Hemoglobin [g/L]	Male	124 (119–137)	n.s	**134 (123–145)**	** < 0.001**	**156 (139–161)**
	Female	116 (112–136)	n.s	124 (122–134)	0.063	133 (128–137)
MCV [fL]	Male	**89.0 (86.4–91.3)**	** < 0.001**	**90.8 (89.4–94.6)**	**0.022**	**89.6 (88.1–92.1)**
	Female	**91.2 (87.5–93.3)**	**0.017**	**93.5 (91.7–93.9)**	**0.018**	**88.5 (86.4–90.0)**
MCH [pg]	Male	30.9 (30.2–31.5)	n.s	**30.5 (29.8–31.6)**	**0.018**	**31.1 (30.8–31.6)**
	Female	30.8 (29.3–31.9)	n.s	**30.9 (29.9–32.0)**	**0.028**	**29.9 (28.1–31.4)**
Lymphocytes [% of WBC]	Male	**15.8 (10.0–23.9)**	**0.001**	**25.2 (21.0–29.8)**	**0.027**	**31.6 (26.7–35.2)**
	Female	10.7 (9.4–34.3)	n.s	29.4 (18.8–33.3)	n.s	31.6 (25.4–35.0)
CRP [mg/dL]	Male	**9.41 (3.5–17.06)**	** < 0.001**	**0.20 (0.06–0.56)**	n.s	0.15 (0.06–0.30)
	Female	**3.42 (1.94–21.53)**	**0.018**	**0.13 (0.08–0.63)**	n.s	0.25 (0.09–0.82)
IL-6 [ng/L]	Male	**85.8 (32.6–205.4)**	** < 0.001**	**3.1 (1.5–7.3)**	n.s	1.5 (1.5–4.3)
	Female	**28.5 (12.3–171.8)**	**0.012**	**3.2 (1.5–6.5)**	0.068	1.5 (1.5–2.6)
LDH [U/L]	Male	**352 (252–400)**	** < 0.001**	**206 (193–224)**	n.s	195 (171–220)
	Female	314 (238–382)	0.093	221 (213–251)	n.s	201 (189–238)
Total serum iron [µmol/L]	Male	**4.2 (3.1–5.7)**	** < 0.001**	**15.4 (8.7–20.1)**	n.s	15.2 (12.8–21.0)
	Female	**4.3 (2.4–15.8)**	**0.012**	**16.3 (11.4–17.7)**	n.s	14.0 (8.0–18.2)
Ferritin [µg/L]	Male	**1420 (572–2053)**	** < 0.001**	**280 (137–469)**	**0.004**	**160 (112–272)**
	Female	**632 (469–973)**	**0.018**	**193 (91–305)**	0.063	91 (50–170)
Transferrin saturation [%]	Male	**13.0 (10.0–17.0)**	**0.004**	**26.0 (13.0–37.0)**	n.s	24.0 (20.0–32.0)
	Female	10.0 (9.0–33.0)	0.063	25.5 (19.0–31.0)	n.s	22.0 (12.0–33.5)

Median values and IQR of study parameters and longitudinal comparison between groups.

*p value comparing parameters during acute COVID-19 infection (acute) and first follow-up (FU1); **p-value comparing parameters at FU1 and FU2; bold indicates significant differences; no significant difference (n.s.). white blood count (WBC), mean corpuscular volume (MCV), mean corpuscular hemoglobin (MCH).

Perturbations of IFN-γ mediated parameters between acute COVID-19 infection and FU1 have already been discussed in a previous publication^27^, therefore we herein only focus on FU2 results and dynamics:

In the following only the most important findings will be summarized, the medians and p-values for all the following results are shown in Table 2.

Regarding IFN-γ mediated parameters only few and rather slight changes between FU1 and FU2 were seen: In women, a further decrease at FU2 compared to FU1 was significant for Kyn/Trp. Interestingly, nitrite was elevated during acute infection and at FU1, while decreasing significantly between FU1 and FU2.

At the two follow-ups a high proportion of patients still had elevated levels of IFN-γ dependent laboratory values exceeding the 95th percentile of healthy blood donors (Geisler et al^41^., see Table 3). Most notably, 69.6% of patients for Kyn/Trp and 65.2% of patients for Phe/Tyr at FU2 respectively had values above the 95th percentile.

**Table 3:** Number of patients exceeding the 95th or 5th percentile of IFN- γ dependent laboratory values during acute COVID-19 infection and at the two follow-ups (FU1, FU2).

	Acute (n = 33)	FU1 (n = 34)	FU2 (n = 23)	95th/5th percentile*
	n (valid %)	n (valid %)	n (valid %)	
Neopterin [nmol/L]	32 (97)	18 (53)	6 (26.1)	9.10^a^
Kyn [µmol/L]	10 (30.3)	11 (32.4)	11 (47.8)	2.48^a^
Trp [µmol/L]	26 (78.8)	17 (50)	0 (0)	53.1^b^
Phe [µmol/L]	31 (93.9)	8 (23.5)	11 (47.8)	83.4^a^
Tyr [µmol/L]	10 (30.3)	16 (47.1)	1 (4.4)	59.5^b^
Kyn/Trp [µmol/mmol]	29 (87.9)	25 (73.5)	16 (69.6)	35.1^a^
Phe/Tyr [µmol/µmol]	33 (100)	28 (82.3)	15 (65.2)	0.95^a^

n, number of patients; valid %, percentage of patients excluding missing values; *according to Geisler et al^41^.; ^a^95th percentile; ^b^5th percentile.

B vitamin (folate and B12) availability, which was decreased at FU1 in men, improved from FU1 to FU2: Folate was significantly higher again in male patients at FU2 compared to FU1. Also, hemoglobin improved significantly in men at FU2 compared to FU1.

We observed a further reduction of ferritin values in male and female patients at FU2 compared to FU1. Other iron metabolism markers showed a return to baseline approximately within the first 60 days after symptom onset (FU1) and did not change afterwards, which is in line with previous observations on subjects with COVID-19 induced anemia^45^.

Inflammatory markers such as CRP and IL-6 were significantly higher during acute illness in both men and women compared with FU2. LDH was significantly elevated only in male patients during acute infection and declined to normal values until FU2.

### Relationship between laboratory parameters and symptoms

Interestingly, fatigued patients at FU2 had higher folate levels than non-fatigued patients (10.8 µg/L vs. 7.7 µg/L; *p* = 0.029), while patients with sleeping problems tended to have higher Kyn (2.7 µmol/L vs. 2.3 µmol/L; *p* = 0.065), higher Kyn/Trp (40.3 µmol/mmol vs. 36.7 µmol/mmol; *p* = 0.086) and also higher folate concentrations (10.4 µg/L vs. 7.0 µg/L; *p* = 0.093) than patients with no problems sleeping. If neurological symptoms were still reported at FU2, patients tended to have lower neopterin levels (6.3 nmol/L vs. 8.0 nmol/L; *p* = 0.088), as well as lower Kyn values (2.1 µmol/L vs. 2.5 µmol/L; *p* = 0.082) compared to patients without neurological symptoms.

### Comparison of acute study parameters disease severity-stratified

We examined laboratory parameters during acute COVID-19 infection depending on disease severity. Again, the most important findings will be summarized, medians and p-values are shown in Table 4.

**Table 4:** Comparison of study parameters during acute COVID-19 infection stratified by disease severity (only significant differences and statistical trends shown).

	Disease severity			
	Moderate (n = 3)	Severe (n = 11)	ICU (n = 20)	*p*-value*
	Median (IQR)	Median (IQR)	Median (IQR)	
Neopterin [nmol/L]	10.1 (7.7–18.3)	42.5 (18.7–46.2)	25.7 (21.2–45.7)	0.051
Kyn [µmol/L]	**1.9 (1.5–1.9)**	**3.7 (3.2–4.4)**	**3.1 (2.5–3.9)**	**0.017**
Kyn/Trp [µmol/mmol]	44.7 (27.7–57.3)	78.8 (63.2–106.7)	61.4 (50.6–89.6)	0.063
Nitrite [µmol/L]	**13.8 (13.3–44.0)**	**13.8 (8.2–22.9)**	**33.2 (19.5–60.5)**	**0.017**
WBC [G/L]	**6.3 (4.3–6.7)**	**5.2 (2.7–6.5)**	**7.7 (5.3–10.0)**	**0.032**
Platelet count [G/L]	**295 (161–306)**	**151 (112–218)**	**250 (195–302)**	**0.039**
ANC [G/L]	**3.4 (2.1–4.3)**	**3.1 (1.8–4.5)**	**6.3 (4.2–8.4)**	**0.004**
Segmented neutrophils [%]	**54.3 (48.9–56.0)**	**62.8 (55.4–66.0)**	**81.6 (76.1–89.4)**	**0.002**
Monocytes [%]	**9.4 (7.3–14.1)**	**9.0 (5.0–15.0)**	**4.6 (2.8–7.4)**	**0.037**
Lymphocytes [% of WBC]	**35.5 (26.3–41.9)**	**23.9 (16.5–27.9)**	**11.2 (7.5–15.4)**	**0.002**
Lymphocytes [G/L]	**2.00 (1.80–2.24)**	**0.95 (0.52–1.67)**	**0.93 (0.72–1.06)**	**0.023**
Fibrinogen [mg/dL]	**453 (239–542)**	**514 (361–592)**	**597 (554–678)**	**0.044**
CRP [mg/dL]	**1.94 (0.06–2.09)**	**3.50 (2.43–9.41)**	**16.06 (8.05–22.09)**	**0.003**
PCT [µg/L]	**0.06 (0.06–0.06)**	**0.08 (0.07–0.33)**	**0.26 (0.13–0.75)**	**0.012**
IL-6 [ng/L]	**15.3 (2.3–41.4)**	**41.6 (20.0–118.1)**	**130.3 (37.3–313.2)**	**0.028**
Troponin T [ng/L]	**5.0 (5.0–8.2)**	**15.5 (10.5–19.2)**	**11.4 (7.8–15.5)**	**0.020**
NT-proBNP [ng/L]	**68 (66–77)**	**443 (208–508)**	**138 (82–464)**	**0.044**
Total serum iron [µmol/L]	**17.5 (4.6–19.4)**	**4.8 (3.9–5.7)**	**3.7 (2.3–4.4)**	**0.011**

Median values and IQR of blood values acquired during acute COVID-19 infection stratified by disease severity.

Significant values are in [bold].

*p-value comparing blood values among groups of disease severity.

Kyn, nitrite, white blood cells (WBC), platelet count, absolute neutrophil count (ANC), segmented neutrophils, monocytes, lymphocytes in percentage of WBC, lymphocyte count, fibrinogen, CRP, PCT, IL-6, troponin T, NT-proBNP, and total serum iron (TSI) differed depending on disease severity.

At FU1, vitamin B12, creatinine, WBC, MCH, platelet count, ANC, CRP, PCT and CK differed significantly among the three categories of disease severity (medians and p-values see Table 5). Neither tryptophan catabolism nor other IFN-γ mediated biochemical pathways differed according to the prior disease severity at FU1. However, about one year after symptom onset (at FU2), patients with a severe course of disease presented with significantly higher levels of Kyn/Trp than patients who had been admitted to the ICU during acute illness (40.3 vs. 28.9; *p* = 0.019).

**Table 5:** Comparison of study parameters at FU1 stratified by disease severity (only significant differences shown).

	Disease severity			
	Moderate (n = 3)	Severe (n = 11)	ICU (n = 20)	*p*-value*
	Median (IQR)	Median (IQR)	Median (IQR)	
Vitamin B12 [pmol/mL]	263 (251–334)	395 (234–717)	229 (188–326)	0.048
Creatinine [mg/dL]	0.81 (0.79–0.90)	0.95 (0.83–1.03)	0.75 (0.70–0.78)	0.020
WBC [G/L]	6.3 (5.3–6.7)	5.7 (4.0–6.9)	7.3 (6.3–9.3)	0.023
MCH [pg]	30.4 (29.8–31.5)	31.7 (31.0–32.7)	30.3 (29.5–30.7)	0.015
Platelet count [G/L]	259 (244–319)	212 (192–238)	303 (226–315)	0.035
ANC [G/L]	3.7 (2.8–5.1)	3.7 (2.2–4.1)	4.3 (3.8–5.5)	0.036
CRP [mg/dL]	0.06 (0.06–0.15)	0.07 (0.06–0.22)	0.36 (0.13–1.06)	0.019
PCT [µg/L]	0.06 (0.06–0.06)	0.06 (0.06–0.06)	0.06 (0.06–0.08)	0.045
CK [U/L]	54 (53–126)	61 (54–121)	38 (31–57)	0.039

Median values and IQR of blood values acquired at FU1 stratified by disease severity.

*p-value comparing blood values among groups of disease severity.

### Comparison of acute study parameters ECOG-stratified

In addition to disease severity, we explored whether patients’ routine laboratory parameters differed regarding ECOG performance status. Due to the partially small sample size, three ECOG groups were formed and subsequently compared during acute COVID-19 infection. An ECOG score of 1 or lower was considered low, ECOG of 2 or 3 was considered medium and an ECOG of 4 was considered high. Nitrite (*p* = 0.022), GOT (p = 0.035), ANC (*p* = 0.038), segmented neutrophils (*p* = 0.011), lymphocytes in percentage of WBC (*p* = 0.008), fibrinogen (*p* = 0.004), CRP (*p* = 0.004), PCT (*p* = 0.047), IL-6 (*p* = 0.016), LDH (*p* = 0.006), and ferritin (*p* = 0.001) differed significantly among the three groups.

At the time of the two follow-ups most patients reported an ECOG-score of 0 or 1. Subsequently we decided to compare these two groups regarding FU1 and FU2. At the time of FU1 hemoglobin levels were higher in patients with an ECOG of zero (138 g/L vs. 125 g/L, *p* = 0.033), while ANC (3.7 G/L vs. 5.0 G/L, *p* = 0.045), CRP (0.08 mg/dL vs. 0.39 mg/dL, *p* = 0.001), IL-6 (1.5 ng/L vs. 4.9 ng/L, *p* = 0.005), LDH (204 U/L vs. 222 U/L, *p* = 0.014), troponin T (8.1 ng/L vs. 19.5 ng/L, *p* < 0.001), and NT-proBNP (109 ng/L vs. 396 ng/L, *p* < 0.001) were lower compared with an ECOG score of one.

The statistical comparison at FU2 showed higher levels for Phe and Tyr, as well as hepcidin25, MCH, and ferritin in patients with a lower ECOG score. LDH and transferrin showed inverse results (see medians and p-values in Table 6).

**Table 6:** Comparison of blood values collected at FU2 ECOG-stratified (only significant differences and statistical trends shown).

	ECOG FU2		
	0	1	*p*-value*
	(n = 14)	(n = 13)	
	Median (IQR)	Median (IQR)	
Phe [µmol/L]	**88.2 (84.1–94.2)**	**73.4 (68.2–77.3)**	**0.036**
Tyr [µmol/L]	**88.0 (68.8–93.0)**	**74.7 (63.8–80.6)**	**0.043**
25-OH-Vit D3 [nmol/L]	**48 (40–69)**	**85 (76–106)**	**0.008**
Hepcidin-25 [μg/L]	**13.6 (8.2–28.0)**	**5.1 (2.6–13.9)**	**0.009**
MCH [pg]	**31.6 (31.2–32.0)**	**31.0 (28.6–31.1)**	**0.002**
Platelet count [G/L]	219 (165–253)	273 (184–288)	0.076
LDH [U/L]	**186 (171–195)**	**216 (197–259)**	**0.022**
Ferritin [µg/L]	**216 (131–293)**	**102 (42–143)**	** < 0.001**
Transferrin [mg/dL]	**248 (216–274)**	**275 (250–318)**	**0.019**

Median values and IQR of blood parameters collected at FU2 ECOG-stratified.

*p-value comparing blood values among patients with an ECOG score of 0 and 1 at FU2, bold indicates significant differences; no significant difference (n.s.).

### Correlation of study parameters

We further assessed correlations between ECOG-score, disease severity, and routine laboratory parameters each during acute COVID-19 infection and at FU1 and FU2 respectively.

High vitamin B12 levels during acute COVID-19 infection correlated significantly with lower Trp (rs = − 0.677; *p* = 0.001), and higher Kyn/Trp (rs = 0.504; *p* = 0.024).

Patients with a more severe disease (rs =− 0.381; *p* = 0.034) or a higher ECOG score (rs =− 0.417; *p* = 0.022) had lower levels of vitamin B12 at the time of FU1. Furthermore, higher CRP levels at FU1 coincided with lower folate levels at FU1 (rs =− 0.374; *p* = 0.046). High folate was also significantly related to lower Trp (rs = − 0.605; *p* < 0.001) as well as lower Phe (rs = − 0.470; *p* = 0.01) at FU1.

At FU2 patients with a more severe course of acute COVID-19 infection presented with tendentially lower folate (rs = − 0.385; *p* = 0.058) and lower vitamin B12 concentrations (rs = − 0.341; *p* = 0.07).

## Discussion

Since the 5th of May 2023 the World Health Organization no longer declares COVID-19 as a global health emergency. However, SARS-CoV-2 virus is endemic and will continue to challenge the public, as well as the scientific community. Research into the molecular basis of disease trajectory and Long-COVID is therefore essential. In view of that, we investigated IFN-γ induced parameters and routine laboratory values, as well as disease severity and ECOG score during acute COVID-19 infection and over the course of one year after symptom onset.

As expected, ECOG performance status was significantly higher during acute COVID-19 infection compared to the two follow ups, indicating that acute infection strongly impaired physical performance. Affirmatively, this was widely reported in previous studies^7,46^. At the time of FU1 most patients had recovered a physical constitution within the range of ECOG 0–1, after one year about half of the patients were fully recovered and able to perform as before. Still, about half of the patients reported an impaired ability to work (ECOG 1) and presented with persistent symptoms after one year.

As metabolic changes induced by the virus and/or over-whelming immune response might be connected with the development of symptoms, we wanted to investigate the dynamics of different biochemical pathways, that have earlier been associated with symptoms like fatigue, depression or impaired quality of life. Furthermore, it was our goal to examine, whether disease severity influenced the investigated pathways long-term and whether patients with symptoms differed regarding the investigated lab parameters or not.

Numerous laboratory parameters showed notable differences between acute COVID-19 infection and follow-up investigations: Inflammatory parameters decreased within a standard range by the first follow-up, both in women and men. Interestingly, there appeared to be differences regarding immune response already during acute infection: Female patients presented with lower median concentrations of inflammatory markers CRP and IL-6 during acute illness, but higher neopterin values (also at FU1, at FU2 median concentrations of all the three inflammatory markers were within normal ranges in both men and women).In the past, the role of the X-chromosome among others was discussed as a possible reason for sex differences in immune regulation^47,48^. Male patients presented with low lymphocytes during acute infection, which steadily and significantly increased at FU1 and FU2, which is also consistent with earlier findings^7,49,50^.

Female patients were suffering more frequently than men from neurological symptoms, fatigue and sleep disturbance a year after infection which is supported by related research^51^. We cannot really explain the finding, that women did not report about neurological symptoms at FU1, while many reported them after 1 year: maybe deficits were regarded by women as mild and reversible initially, but in the course of reconvalescence they impaired everyday life strong. Another explanation might be that symptoms developed thereafter, which has also been stated by many other female patients who had a mild course of disease and later on developed symptoms of Long Covid.

Our data also fit well with results of an earlier study showing that men are more severely impacted in the acute phase, since mortality is higher in men^52^, and women are more likely to suffer long-term consequences.

In total, we observed a return to normal ranges regarding most investigated parameters by FU2, with negligible changes between FU1 and FU2 in our patient cohort. However, surprisingly, IFN-γ dependent laboratory values were still elevated one year after infection in a substantial number of patients. About two thirds of our cohort had values for Kyn/Trp (69.6%) and Phe/Tyr (65.2%) exceeding the 95th percentile compared to healthy adults. Thus, it seems that moderate to severe COVID-19 infection impairs IFN-γ mediated pathways up to one year after the onset of symptoms, which has not been previously reported and adds to our knowledge.

Moreover, there also appears to be a link between immune-mediated biochemical changes and clinical symptoms in our study. Patients, whose physical performance had returned to normal function, presented with higher concentrations of iron metabolism parameters (ferritin, transferrin, hepcidin) and amino acids (phenylalanine and tyrosine) than those with impaired ability to work and function (ECOG 1). Patients with sleeping problems tended to have higher Kyn and higher Kyn/Trp, while patients with fatigue had (rather unexpectedly) higher folate concentrations. Overall, the association of clinical symptoms with lab parameters influenced by IFN-γ signaling at FU2 appears to be more complex and individual, since patients with neurological symptoms at FU2 tended to have lower neopterin and Kyn concentrations.

Also B vitamin metabolic changes appeared to be associated with tryptophan catabolism (both during acute infection and at FU) and disease severity: our data support a relationship between vitamin B12 and COVID-19 severity, which was already found in other studies^38,53^. Patients with a more severe disease and a continued higher impairment of their physical functioning ability had lower vitamin B12 levels at FU1 (and high MCV levels), indicating enhanced vitamin B12 demand during acute infection (probably as compensatory mechanism, reflected by high B12 levels in men) and lower availability during reconvalescent COVID-19 infection. Folate concentrations were interestingly also associated with tryptophan metabolism at FU1 and related with fatigue at FU1.

Thus, our data indicate that metabolic changes are related with each other and with symptoms in reconvalescent patients. The small cohort size and a relative predominance of severe cases are major limitations which have to be taken into account and which restrict the generalizability of our results. On the other hand, the fact that the laboratory parameters were not influenced by immuno-suppressive treatment with e.g., corticoids (like in the later course of the pandemic) is a strength of this study, as immune-mediated biochemical changes could be monitored in a small, but well defined study population. Conclusively, similar investigations in larger cohorts of Long Covid patients are certainly needed to better extrapolate, whether and how exactly dysbalances of these biochemical pathways are really contributing to the persistence of symptoms in patients with Long Covid or not.

## Data Availability

Results presented within this study are available within the manuscript. Blinded raw data are available on demand.
